# Supplementary figures and images for: Functional Dynamics and Selectivity of Two Parallel Corticocortical Pathways from Motor Cortex to Layer 5 Circuits in Somatosensory Cortex
Source: eNeuro. 2024 Jun 14;11(6):ENEURO.0154-24.2024. doi: 10.1523/ENEURO.0154-24.2024 (PMC11209671; doi:10.1523/ENEURO.0154-24.2024)

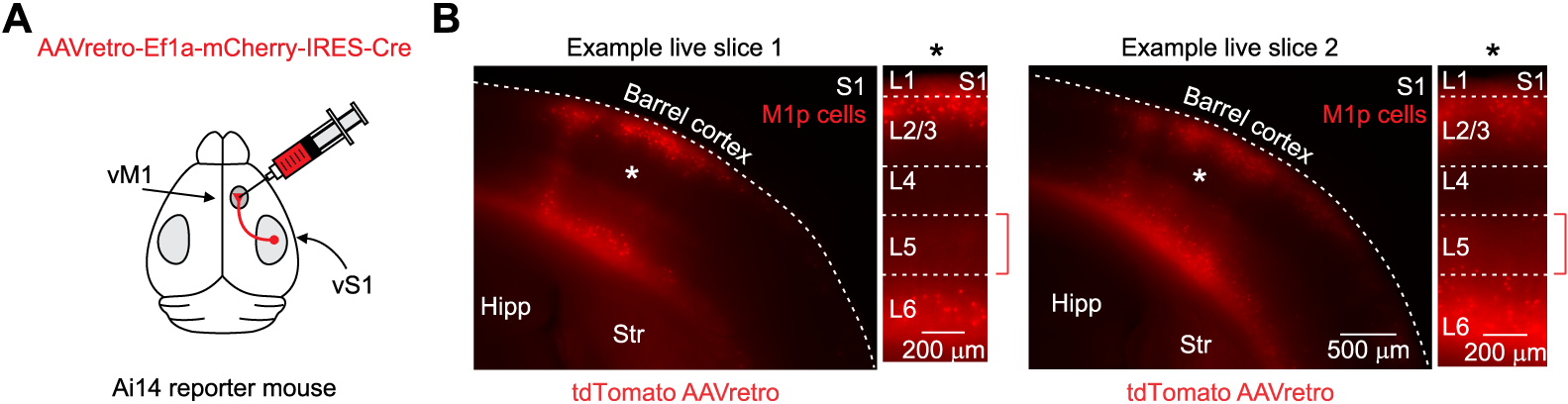

Supplement: Figure 1-1 — Anatomical characterization of L5 IT neurons labeled with AAVretro. A, Injection schematic showing AAVretro carrying genes for Cre and mCherry was injected unilaterally into M1 of Ai14 mice in vivo at ∼3 weeks of age. B, Two example fluorescent images of live coronal slices (300 μm thick) through S1 from two different Ai14 mice injected in M1 ∼21 days prior with AAVretro.EF1a-mCherry-IRES-Cre. Images show tdTomato expressing S1 neurons following AAVretro injection. Higher magnification images show retrogradely labeled neurons in L2/3 and L6, but very few in L5 (n = 5 mice). Download Figure 1-1, TIF file. [file eneuro-11-ENEURO.0154-24.2024-s001.tif]
